# A230 A MISSENSE MUTATION IN GOBLET CELL PROTEIN FCGBP ALTERS THE GLYCOMIC PROFILE AND FUNCTION OF THE COLONIC MUCUS BARRIER

**DOI:** 10.1093/jcag/gwac036.230

**Published:** 2023-03-07

**Authors:** H Gorman, F Moreau, W Zandberg, K Bergstrom, K Chadee

**Affiliations:** 1 Microbiology, Immunology and Infectious Diseases, University of Calgary, Calgary; 2 Chemistry; 3 Biology, University of British Columbia Okanagan Campus, Okanagan, Canada

## Abstract

**Background:**

MUC2 mucin, produced by colonic goblet cells, forms a mucus bilayer that provides a physical barrier between potential pathogens in the lumen and the underlying epithelial cells. Mucus is thus the first line of innate host defense in the gastrointestinal (GI) tract. Many GI diseases including inflammatory bowel disease and colon cancer affect the glycosylation of mucus. Goblet cells produce a variety of proteins that are associated with the mucus layer. Of these proteins, FCGBP is of significant interest due to its structural similarities to MUC2 mucin with unknown functions. In this study, we investigated how a missense mutation in FCGBP altered the glycosylation of goblet cell MUC2 and affected its barrier functions.

**Purpose:**

Hypothesis: A missense mutation in FCGBP results an impaired mucus layer by the altering glycomic profiles of goblet cell mucins. Specific aims:

1) To determine mechanistically how FCGBP impeded the structural integrity of the mucus layer

2) To quantify MUC2 glycoprotein modifications in the altered mucus layer

**Method:**

To investigate whether FCGBP impaired mucus barrier functions, two cell types were investigated: wildtype LS174T (WT) MUC2 mucus-producing goblet cells and LS174T cells with a missense mutation in FCGBP (FCGBP MS). To determine if FCGBP MS led to loss in barrier function in the mucus layer, the penetration of 0.2, 1, and 2 μm fluorescent beads (to mimic bacteria) through the mucus layer were quantified. To determine if the differences in penetrability were caused by differences in MUC2 glycosylation in the goblet cell lines, sensitive glycomic analyses were performed by high-performance liquid chromatography-mass spectrometry (HPLC-MS) and capillary electrophoresis with laser-induced fluorescence detection (CE-LIF). Both intact cells and isolated MUC2 mucin granules were analyzed. To determine if differences in the glycomic profiles was caused by differences in glycotransferases, RT-PCR was performed on over 30 human glycosyltransferases.

**Result(s):**

FCGBP MS cells exhibited significant loss in MUC2 mucus barrier function as quantified by fluorescent beads penetration through the mucus layer in a temporal manner. FCGBP MS cells exhibited an altered glycomic profile with a notable increase in sialylated glycans as quantified by HPLC-MS. The increase in sialylated glycans was associated with a significant increase in sialyl-transferase expression in FCGBP MS cells.

**Conclusion(s):**

These data demonstrate that a single missense mutation in FCGBP altered the penetrability of the mucus layer associated with an increase in sialylated proteins, a signature hallmark of numerous colonic diseases. FCGBP was critical in providing structural integrity of the mucus layer and maintenance of goblet cell glycosylation profiles.

**Please acknowledge all funding agencies by checking the applicable boxes below:**

CIHR

**Disclosure of Interest:**

None Declared

